# Taxonomic review of the *Themus* (*Telephorops*) *nepalensis* species-group (Coleoptera, Cantharidae)

**DOI:** 10.3897/zookeys.884.32550

**Published:** 2019-10-30

**Authors:** Yuxia Yang, Huacong Xi, Xingke Yang, Haoyu Liu

**Affiliations:** 1 The Key Laboratory of Zoological Systematics and Application, College of Life Sciences, Hebei University, Baoding 071002, Hebei Province, China Hebei University Baoding China; 2 Key Laboratory of Zoological Systematics and Evolution, Institute of Zoology, Chinese Academy of Sciences, Beijing 100101, China Institute of Zoology, Chinese Academy of Sciences Beijing China

**Keywords:** female internal genitalia, new faunistic record, new synonym, soldier beetles, taxonomy

## Abstract

The diagnosis of the Themus (Telephorops) nepalensis species-group is summarized. A catalogue, a key and a distribution map of all world species are provided. Two synonymies are proposed: Themus (Telephorops) subcaeruleiformis Wittmer, 1983, **syn. nov.** = T. (Telephorops) crassimargo Champion, 1926; T. (Telephorops) separandus Wittmer, 1975, **syn. nov.** = T. (Telephorops) laboissierei (Pic, 1929). The female internal genitalia are photographed and described in this species-group for the first time, the aedeagi of T. (Telephorops) crassipes Pic, 1929 and T. (Telephorops) impressipennis (Fairmaire, 1886) are illustrated and described for the first time, and some additional distribution information is provided for the species. Themus (Telephorops) cavipennis (Fairmaire, 1897) is a new record for the Chinese fauna.

## Introduction

*Themus* Motschulsky, 1858 is one of the largest cantharid genera and comprises about 250 species in total (Yang et al. 2014; Kopetz 2016). It consists of four subgenera (Wittmer 1973, 1997), which were redefined by Švihla (2008) on the basis of the shapes and color of the pronotum and elytra.

The subgenus Telephorops Fairmaire, 1886 for *T. impressipennis* (by original and monotypic designation) was subdivided into two species groups, which however were not given names (Švihla 2008). According to the Principle of Priority (ICZN 1999, Articles 23.1 and 23.3.3), the valid name of a taxon is the oldest available name applied to it, so the earliest-named member of an aggregate of vicarious species will be the species-group name. They have been named the *davidis* species-group (Yang et al. 2019) and the *nepalensis* species-group (including the type species for the genus) respectively. The latter was characterized by the reduced and shortened laterophyses of the aedeagus and the enlarged elytra with depressions (Švihla 2008).

Most species of the *nepalensis* species-group were described by early taxonomists, such as Fairmaire (1886, 1897), Hope (1831), Pic (1911, 1912, 1926, 1929a,b), Gorham (1889) and Champion (1926). Recently, those species were revised and more species were added by Wittmer (1954, 1975, 1983a, b, 1997). A few more species and additional morphological or distributional information were added by Okushima (1999, 2003), Kopetz (2004, 2010, 2016) and Yang et al. (2013).

Up to now, 15 species were included in the *nepalensis* species-group. This group has not previously been reviewed globally, and some sibling species remain difficult to diagnose from others due to there being few characters known for the females when males are unavailable. Furthermore, attribution of species to the groups is difficult because species diagnoses are often imprecise. For example, *T. minor* Wittmer, 1997, *T. subcaeruleus* (Pic, 1911) and *T. crassimargo* Champion, 1926, whose elytra are enlarged and laterophyses well developed, give contradictory information about their placement in the species-group defined by Švihla (2008). Thus, in the present study, all known species are reviewed to evaluate morphological evidence supporting species groups, and both are redefined where necessary.

## Material and methods

The material is deposited in the following collections:

**BMNH**British Museum of Natural History, London, UK;

**CAUB**Chinese Agriculture University, Beijing, China;

**IZAS**Institute of Zoology, Chinese Academy of Sciences, Beijing, China;

**MHBU** Museum of Hebei University, Baoding, China;

**MNHN**Muséum national d’Histoire naturelle, Paris, France;

**NHMB**Naturhistorisches Museum Basel, Switzerland.

Genitalia of both sexes and abdominal sternites VIII of females were dissected and cleared in 10% KOH solution, and female genitalia were stained with hematoxylin. The female internal genitalia is attached to the ventral side of abdominal tergite IX and the vulva opens between the coxites. The dorsal or ventral side of vagina is established according to the tergite IX. The situation of median oviduct opening is on the opposite side of tergite IX and established as the ventral side of vagina. The diverticulum and spermatheca arise from apex of vagina.

Habitus photos were taken using a Leica M205 A stereomicroscope, multiple image layers were stacked using Combine ZM (Helicon Focus 5.3). Line drawings were made using a camera lucida attached to a Nikon SMZ1500 stereomicroscope, then edited in CorelDRAW 12 and Adobe Photoshop 8.0.1. Body length was measured from the anterior edge of the clypeus to the elytral apex and body width across the humeri of elytra. Morphological terminology of female genitalia followed Brancucci (1980). The key to the species was prepared mainly based on the characters of the aedeagus. If the aedeagi of different species were too similar to be described, the female abdominal sternite VIII and internal genitalia were compared; body size and coloration was also referred to when necessary.

In the checklist, valid scientific names and original sources, synonyms and publications for the taxonomical changes, type localities and depositories, additional material information and all distributions were included, as well as additional description or remarks were added if necessary. Complete label data were cited for type specimens, using square brackets “ []” for our remarks and comments, [p] indicating that the following data were machine printed and [h] that they were handwritten, quotation marks to separate data from different labels. A distribution map was prepared using the geographic information system software ArcGIS (ver. 10.2), based literature records and the author's databases of specimens examined for this study.

The specimens were identified based on examination of types if available and original literature. In practice, species were determined mainly by the aedeagus of male, and the females were associated with males based on evidence that they were collected at the same locality and date. Also, the female could be identified by the structure of abdominal sternite VIII, which was useful in species’ recognition and illustrated in the literature by cantharid specialists. For each species, compared with males, the females have smaller eyes, shorter and narrower antennae, simple middle antennomeres, without smooth narrow impressions along the outer edges, wider pronotum and elytra, and only seven abdominal ventrites.

## Taxonomy

### Themus (Telephorops) nepalensis

Taxon classificationAnimaliaColeopteraCantharidae

species-group

5381790C-0E71-5C91-9D38-5F4C20D5C6DF

#### Diagnosis.

Elytra enlarged posteriorly and widest near apical third. Aedeagus: conjoint dorsal plate of parameres narrowed apically in dorsal view, emarginate at middle of apical edge; laterophyses flattened dorsoventrally, reduced and not reaching apices of conjoint dorsal plate except in a few species. Female internal genitalia: diverticulum situated at end of vagina, presenting with a sclerotized ring around at base, confluent in middle and extending to median oviduct; spermatheca arising from middle of the sclerotized ring.

**Figure 1.**
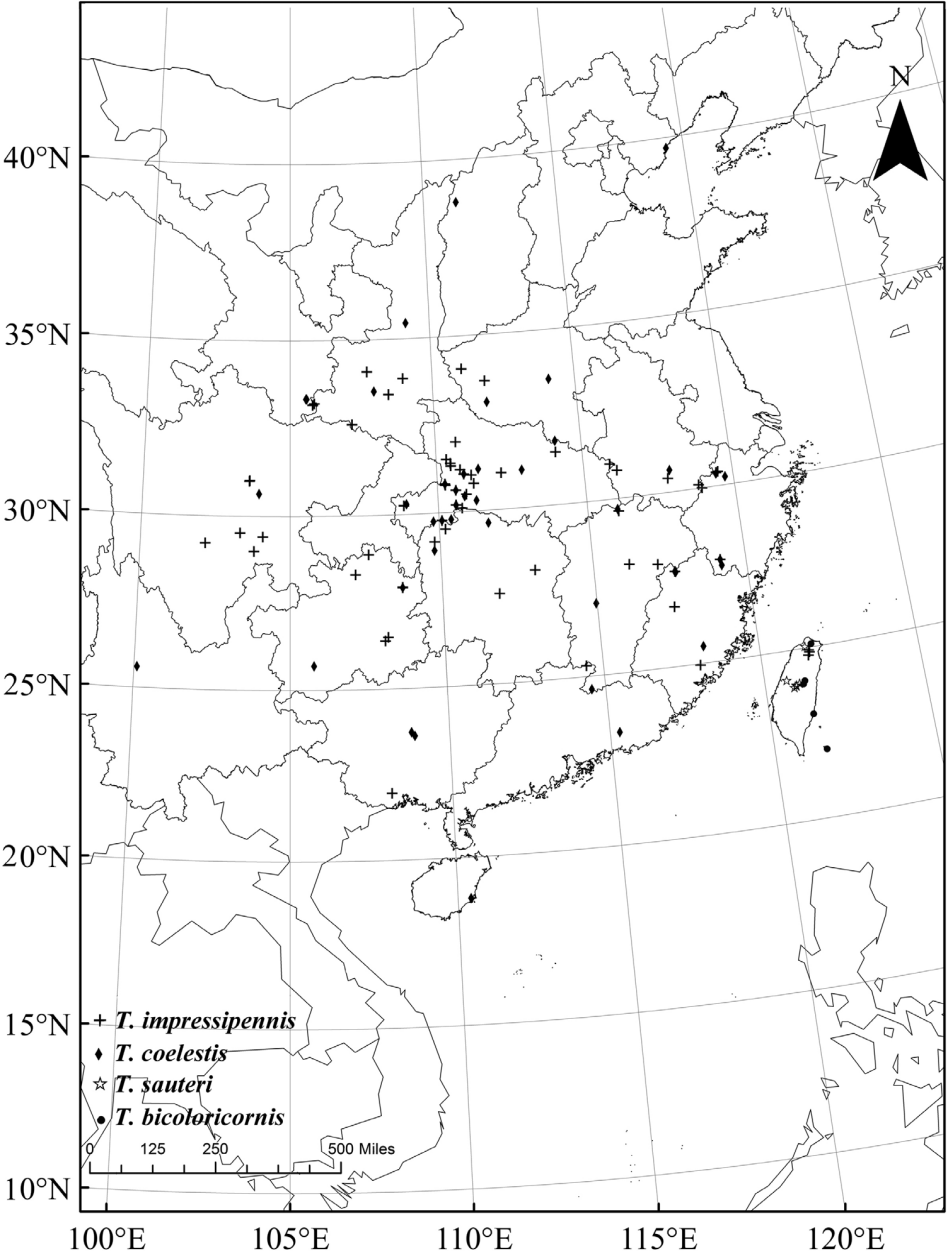
Distribution map of Themus (Telephorops) nepalensis species-group (part I).

**Figure 2.**
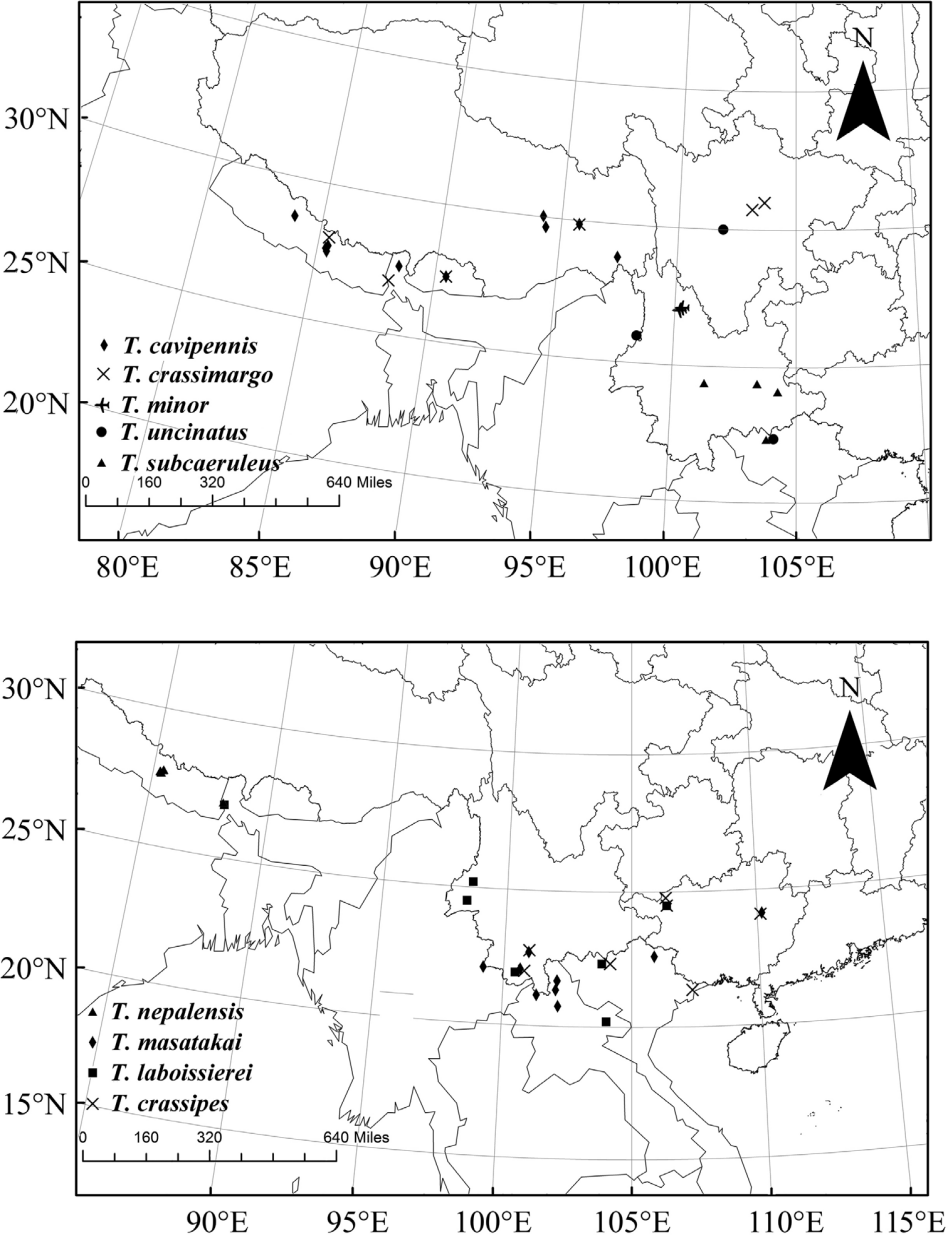
Distribution map of Themus (Telephorops) nepalensis species-group (part II).

**Distribution.** Most species are restricted in their distribution (Figs 1, 2), except *T. impressipennis* (Fairmaire, 1886) and *T. coelestis* (Gorham, 1889), which are widely distributed in China.

#### Remarks.

The diagnosis is developed from the definition of the species-group by Švihla (2008). Characters of the elytra and aedeagus, the female internal genitalia are supplemented in the present study. This differs from the *davidis* species-group in the female genitalia having a sclerotized ring around the base of the diverticulum, delimiting it from the vagina; and spermatheca opening on the opposite side to the median oviduct. While in the *davidis* species-group, there are only a pair of short conjoint sclerotized ridges below the diverticulum, hardly delimitated from the vagina; and spermatheca opening on the same side as the median oviduct (Yang et al. 2019).

### Key to species (adults) of Themus (Telephorops) nepalensis species-group

(characters based on illustrations in the present study or those from Wittmer (1983a, b, 1997)).

**Table:** 

1	Aedeagus: laterophyses reaching apices of conjoint dorsal plate of parameres	**2**
–	Aedeagus: laterophyses reduced, not reaching apices of conjoint dorsal plate of parameres	**4**
2	Antennae, mid and hind legs uniformly black; aedeagus (Wittmer 1997: fig. 104): laterophyses without processes on both sides	***T. minor* Wittmer, 1997**
–	Antennae and legs mixed yellow and black; aedeagus: laterophyses with a narrow process each side	**3**
3	Aedeagus (Wittmer 1983b: fig. 2): conjoint dorsal plate of parameres triangularly emarginate in middle of apical edge in dorsal view; female abdominal sternite VIII (Wittmer 1983b: fig. 62) with lateral protuberances of posterior edge nearly as wide as distance between them	***T. subcaeruleus* (Pic, 1911)**
–	Aedeagus (Wittmer 1983b: fig. 3a): conjoint dorsal plate of parameres rectangularly emarginate in middle of apical edge in dorsal view; female abdominal sternite VIII (Fig. 9D) with lateral protuberances of posterior edge about half as wide as distance between them	***T. crassimargo* Champion, 1926**
4	Aedeagus: ventral process of each paramere hooked at apex in lateral view	**5**
–	Aedeagus: ventral process of each paramere not hooked at apex	**9**
5	Aedeagus: ventral process of each paramere expanded and obtusely hooked dorsally at apex (e.g. Fig. 5C)	**6**
–	Aedeagus: ventral process of each paramere narrowed and acutely hooked ventrally at apex	**8**
6	Elytra dark green or blue, strongly metallic	***T. nepalensis* (Hope, 1831)**
–	Elytra purple-black, weakly metallic	**7**
7	Body longer than 16.0 mm; female internal genitalia (Fig. 6D) with diverticulum narrowed apically	***T. crassipes* Pic, 1929**
–	Body 13.0–15.0 mm in length; female internal genitalia (Fig. 7C) with diverticulum expanded apically	***T. masatakai* Okushima, 2003**
8	Aedeagus (Fig. 6B): ventral process of each paramere triangularly protuberant apicolaterally in dorsal view; female abdominal sternite VIII (Fig. 9B) with each protuberance narrower than the distance between it and apicolateral angle	***T. cavipennis* (Fairmaire, 1897)**
–	Aedeagus (Wittmer 1983b: fig. 5): ventral process of each paramere normal, not protuberant in dorsal view; female abdominal sternite VIII (Fig. 9J) with each protuberance wider than the distance between it and apicolateral angle	***T. uncinatus* Wittmer, 1983**
9	Elytra no more than 1.5 times as long as maximal width; aedeagus (Wittmer 1983b: fig. 4): ventral process of each paramere with apex slightly bent inwards in ventral view, nearly as long as conjoint dorsal plate in lateral view	***T. laboissierei* Pic, 1929**
–	Elytra about twice as long as maximal width; aedeagus: ventral process of each paramere with apex unlike above, not bent inwards in ventral view, longer than conjoint dorsal plate in lateral view	**10**
10	Aedeagus (Wittmer 1983a: fig. 47): ventral process of each paramere abruptly narrowed at apex in ventral view; female abdominal sternite VIII (Fig. 9I) with acute apicolateral angles	***T. sauteri* (Pic, 1912)**
–	Aedeagus (Fig. 6C): ventral process of each paramere expanded at apex in ventral view; female abdominal sternite VIII with rounded apicolateral angles	**11**
11	Tibiae mixed yellow and black; aedeagus (Wittmer 1983b: fig. 1): ventral process of each paramere narrowed apically in ventral view, conjoint dorsal plate widely emarginate medially at apical edge in dorsal view	***T. coelestis* (Gorham, 1889)**
–	Tibiae uniformly black or yellow; aedeagus: ventral process of each paramere almost even in width in ventral view, conjoint dorsal plate narrowly emarginate medially at apical edge in dorsal view	**12**
12	Femora mixed yellow and black, tibiae black; female abdominal sternite VIII (Fig. 9F) with protuberances of posterior edge not reaching apices of apicolateral angles in ventral view	***T. impressipennis* (Fairmaire, 1886)**
–	Femora and tibiae uniformly yellow; female abdominal sternite VIII (Fig. 9A) with protuberances of posterior edge exceeding apices of apicolateral angles in ventral view	***T. bicoloricornis* Wittmer, 1983**

### Themus (Telephorops) bicoloricornis

Taxon classificationAnimaliaColeopteraCantharidae

Wittmer, 1983

83D1908D-B96B-5972-A466-DB3EB2AECBCB

Figs 6A , 9A

Themus (Telephorops) bicoloricornis Wittmer, 1983a: 153, figs 48 (aedeagus illustration), 51 (female abdominal sternite VIII illustration).

#### Type material examined.

1♂ (**paratype**, NHMB), [h] “Idabon \ Musha \ 23.7.1928”, [p] “PARATYPUS”, [h] “Themus (Tryblius) \ bicoloricornis \ Wittm. \ det. W. Wittmer”, [p] “Naturhist. \ Museum Basel \ coll. W. Wittmer”, [p] “CANTHARIDAE \ CANTH00002241”.

**Other material examined.** 1♂, 1♀ (IZAS), Taiwan, Nantou, Tzuei-feng, 1997.VII.9, leg. K. Mizota.

**Supplementary description.** Female. Like male, but antennomeres V–X without impressions along outer edges (while present with smooth narrow longitudinal or oblong impressions in male), terminal abdominal ventrite wide (while narrow and triangular in male) (Fig. 9A) with posterior edge narrowly and triangularly emarginate medially and paired rounded middle protuberances, which are wider than the distance between protuberance and apicolateral angle and exceeding apex of the latter. Internal genitalia (Fig. 6A): diverticulum hardly narrowed apically and rounded at apex, about 2.5 times as long as its maximal width; spermatheca expanded apically.

#### Distribution.

Taiwan.

### Themus (Telephorops) cavipennis

Taxon classificationAnimaliaColeopteraCantharidae

(Fairmaire, 1897)

54F87AE9-1860-5B29-BEAF-2A9B36CB5263

Figs 3A , 6B , 9B

Tryblius cavipennis Fairmaire, 1897: 228.
Themus ancoralis Champion, 1926: 128. Synonymized by Wittmer 1975: 251.
Themus (Tryblius) cavipennis : Pic 1929a: 195; Wittmer 1975: 251, fig. 1 (aedeagus illustration).
Themus (Telephorops) cavipennis : Wittmer 1983b: 197; Okushima 1999: 59, figs 10 (habitus photo), 34 (female abdominal sternite VIII illustration).

#### Type material examined.

1♂ (**holotype**, MNHN), [p]“Himalaya \ Sikkim”, [h]“*Tryblius* \ *cavipennis* \ Fairm., Sikkim”, [p]“HOLOTYPUS”, [h]“*Themus* \ (*Tryblius*) \ *cavipennis* \ Fairm. \ det. W. Wittmer”.

**Other material examined. CHINA**: **Xizang**: 1♂, 1♀ (IZAS), Bomi, Tangmai, 2300m, 2005.VIII.31, leg. X.J. Wang; 1♀ (IZAS), Nyingchi, Pêlong, 2100 m, 2005.IX.2, leg. X.L. Chen; 1♀ (IZAS), same data, 2005.IX.1; 1♀ (IZAS), same locality and date, 2115 m, leg. X.J. Wang; 1♀ (IZAS), Nyingchi, Zayü, Shang Zayü, 1960m, 2005.VIII.23, leg. X.L. Chen; 1♀(IZAS), Zayü, Zhowagoin, Xungjug, 1938 m, 28.6067N, 97.2816E, 2014.VIII.29, leg. H. Liu; 1♀(MHBU), Shang Zayü, 2005.VII.14, leg. A.M. Shi; 1♀(MHBU), Nyingchi, Pêlong, 2007.IX.23.–28, leg. F.M. Shi.

#### Supplementary description.

**Figure 3.**
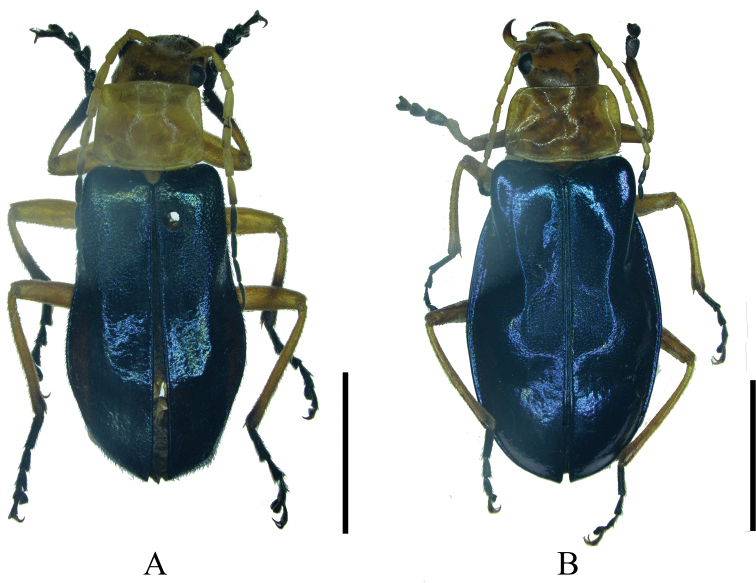
Male habitus, dorsal view **A***Themus cavipennis* Champion, 1926 (the specimen of Xizang) **B***T. crassimargo* Champion, 1926 (the specimen of Sichuan). Scale bars: 5.0 mm.

Male (Fig. 3A). Female. Like male, but antennomeres IV–X without impressions along outer edges (while present with smooth narrow longitudinal or oblong impressions in male), terminal abdominal ventrite wide (while narrow and triangular in male) (Fig. 9B) with posterior edge narrowly and triangularly emarginate medially between paired rounded middle protuberances, each protuberance narrower than the distance between it and apicolateral angle and exceeding apex of apicolateral angle. Internal genitalia (Fig. 6B): diverticulum narrowed apically and nearly pointed at apex, about twice as long as its maximal width; spermatheca abruptly expanded apically.

#### Distribution.

China (new record: Xizang), Bhutan, Nepal, northern India.

**Figure 4.**
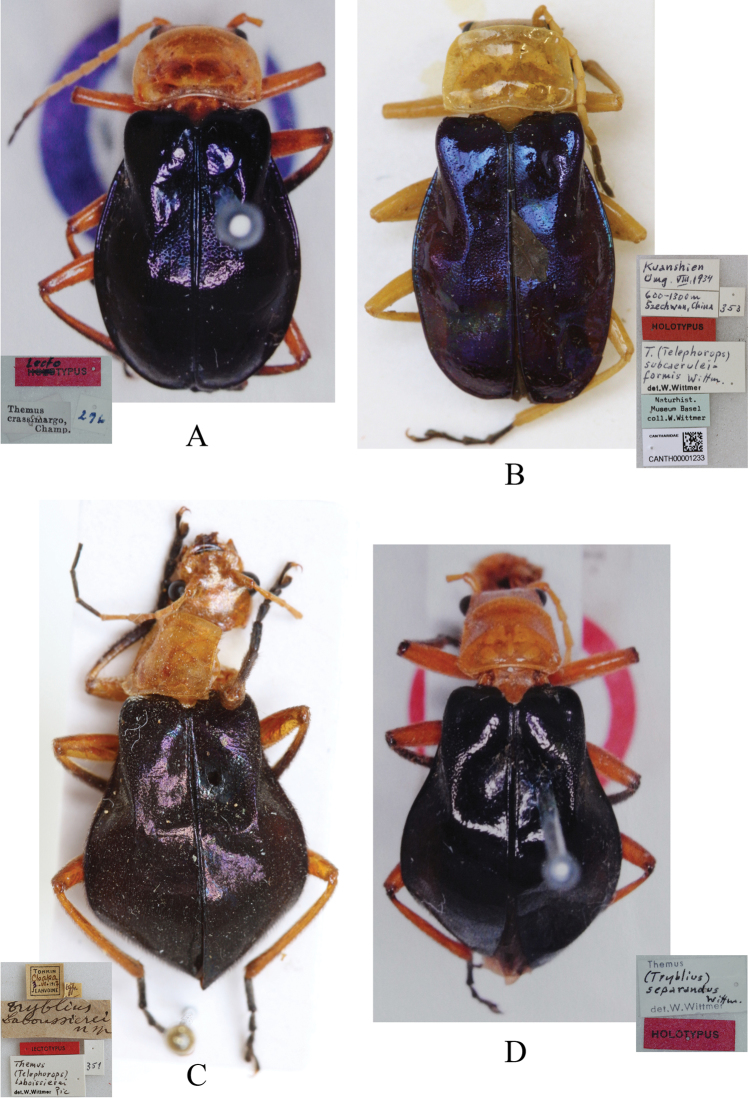
Habitus, dorsal view **A***Themus crassimargo* Champion, 1926 (lectotype) **B**Themus (Telephorops) subcaeruleiformis Wittmer, 1983 (holotype) **C***Triblius laboissierei* Pic, 1929 (lectotype) **D**Themus (Tryblius) separandus Wittmer, 1975 (holotype).

**Figure 5.**
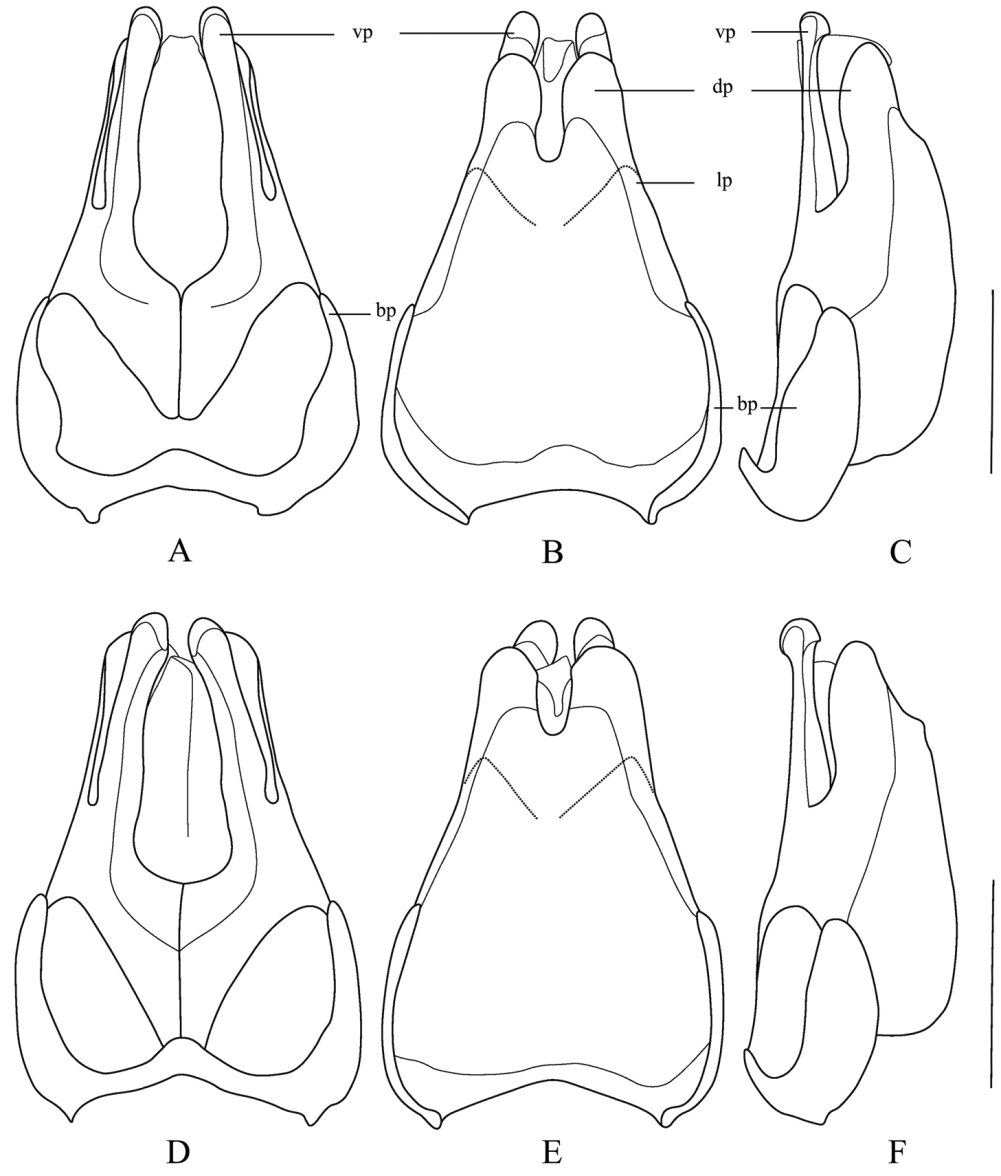
Aedeagus (**A, D** ventral view **B, E** dorsal view **C, F** lateral view) **A–C***Themus crassipes* Pic, 1929 **D–F***T. impressipennis* (Fairmaire, 1886). Scale bar: 1.0 mm. (vp: ventral process of each paramere; dp: conjoint dorsal plate of parameres; lp: laterophyse; bp: basal piece).

### Themus (Telephorops) coelestis

Taxon classificationAnimaliaColeopteraCantharidae

(Gorham, 1889)

B7A0C2C4-6B89-50A2-98B9-9A8271DFAF10

Figs 6C , 9C

Telephorus coelestis Gorham, 1889: 104, t.10, fig. 7.
Themus (Telephorops) coelestis : Wittmer 1983b: 197, figs 1 (aedeagus illustration), 59 (female abdominal sternite VIII illustration).
Themus rugosus Pic, 1929b: 8. Synonymized by Wittmer 1983b: 197.
Themus violetipennis Wang & Yang, 1992: 265, fig. 2 (habitus illustration). Synonymized by Yang et al. 2013: 3.

#### Type material examined.

1♂ (NHMB, **lectotype** of *Telephorus coelestis*): without locality information, [h]“coelestis ♂”, [h]“♂”, [h]“Themus \ (Telephorops) \ coelestis \ (Gorh.) \ det. W. Wittmer”, [h] “Type”, [p] “LECTOTYPUS”, [p]“Naturhist. \ Museum Basel \ coll. W. Wittmer”, [p]“CANTHARIDAE \ CANTH00001277”. The lectotype was designated by Wittmer (1983b).

1♀ (MNHN, **holotype** of *Themus rugosus*), [h]“Fokien” (China, Fujian), [h]“Themus \ rugosus \ n. sp.”, [h]“Themus \ (Telephorops) \ coelestis \ (Gorh.) \ det. W. Wittmer”, [h]“type”, [p]“TYPE”. The holotype is damaged, lacking antennae and right meta-leg.

1♀ (IZAS, **neotype** of *Themus violetipennis*), [p] “Hunan, Yongshun, Shanmuhe forestry station \ 600m”, [p] “4.VIII.1988 \ leg. Shu-Yong Wang”. The neotype was designated by Yang et al. (2013).

#### Other material examined.

**CHINA**, **Shaanxi**: 2♂ (MHBU), Chushui, Niubeiliang, 1056 m, 2011.VIII.22–29, leg. X.C. Zhu & Y. Zhao. **Hubei**: 1♀ (MHBU), Badong, Lvcongpo, 1700 m, 2006.VII.14, leg. M. Li; 1♀ (MHBU), same data, leg. J. H. Wan; 1♀(MHBU), Yuan’an, Hehua, 2009.VII.12, leg. X.M. Sun; 1♀ (MHBU), same data, leg. Y. Dong; 1♀ (MHBU), Yichang, Xianrenxi, 2009.VI.25, leg. G.L. Xie; 1♀ (MHBU), Yichang, Dalaoling Forestry, 2009.VI.26, leg. Y. Tian; 1♀ (MHBU), Yidu, Niejiahe, 2008.VI.16, leg. G. L. Xie; 1♀ (MHBU), Yichang, Hejiaping, Qinggangping, 2013.VII–XI, leg. T.H. Du; 1♀ (MHBU), Changyang, Langping, Changfeng, 900 m, 2013.VII.11, leg. Y.Q. Wu; 1♂ (MHBU), Jingshan, Huzhuashan Forestry, 2007.VII.15, leg. G.L. Xie; 1♂ (MHBU), Wufeng, Houhe, 2002.VII.16, leg. F.Y. Wang; 1♂ (MHBU), same locality, 2002.VII.21, C.H. Shi. **Guangxi**: 1♀ (MHBU), Luocheng, Pingying, 2004.V.29, leg. J.M. Zhang. **Guangdong**: 1♀ (MHBU), Nanling, 2010.VIII.8–18, leg. H.Y. Liu; 1♀ (MHBU), same locality and collector, 2010.VIII.17; 1♀ (MHBU), same locality and collector, 2010.VIII.8–11. **Hebei**: 1♂ (MHBU), Changli, Huangjin seaside, 1999.VIII.18, leg. H.Z. Liang; 1♂ (MHBU), same data, leg. Z.J. Ma; 1♂(MHBU), Zushan, 1998.VII.14, leg. X.J. Li. **Zhejiang**: 1♀ (MHBU), Longquan, Fengyangshan, 2007.VII.25, leg. L.K. Tan; 1♀ (MHBU), same locality and collector, 2007.VII.30; 1♀ (MHBU), same locality, 2007.VII.26, leg. G.L. Xie; 1♀ (MHBU), same locality and collector, 2007.VII.27; 1♀ (MHBU), same locality and collector, 2007.VII.31; 3♂, 1♀ (MHBU), same locality, 2007.VII.25–VIII.1, leg. H.Y. Liu & Z.H. Gao; 1♂ (MHBU), same locality, 2012.VII.18, leg. G.L. Xie & J. Jiao; 2♀ (MHBU), Lin’an, Tianmushan, 2013.VI.26–VII.2, leg. J.Y. Su; 2♀ (MHBU), Hangzhou, Lin’an, Dajingwu, 2012.VI.10, leg. H. Xu; 1♂ (MHBU), Qingyuan, Baishanzu, 2012.VII.24, G.L. Xie & X. Wang. **Yunnan**: 2♂ (MHBU), Dali, 2008.VIII.18, leg. G. L. Xie.

#### Supplementary description.

**Figure 6.**
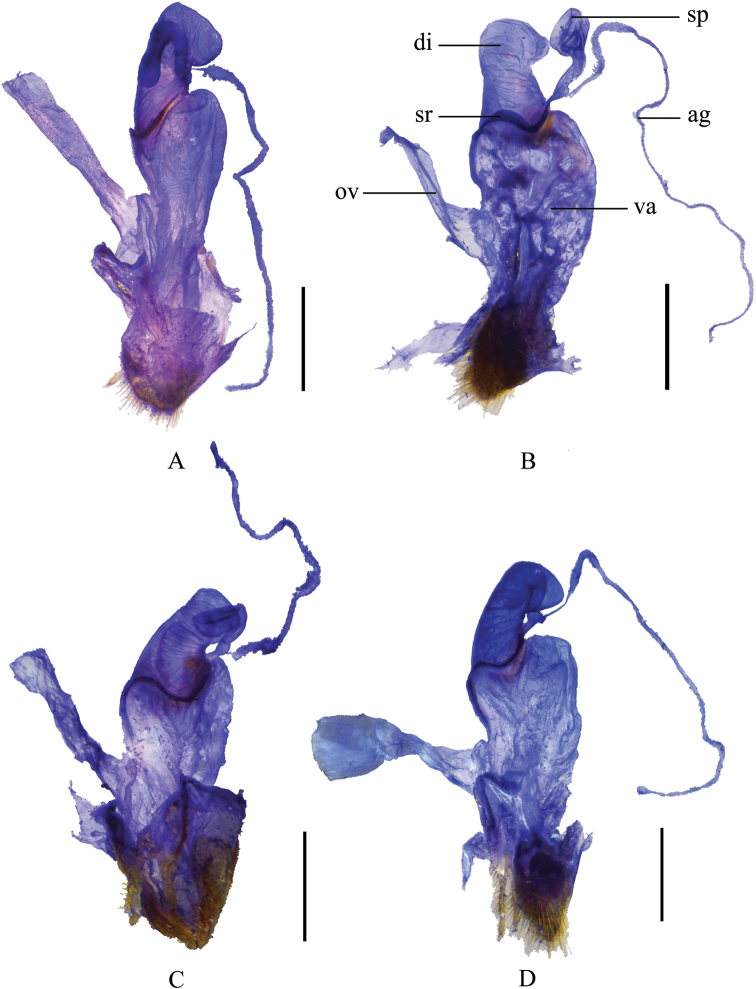
Female internal genitalia, lateral view **A***Themusbicoloricornis* Wittmer, 1983 **B***T. cavipennis* Champion, 1926 **C***T. coelestis* (Gorham, 1889) **D***T. crassipes* Pic, 1929. Scale bars: 1.0 mm.

Female. Like male, but antennomeres IV–X without impressions along outer edges (while present with smooth narrow longitudinal or oblong impressions in male), terminal abdominal ventrite wide (while narrow and triangular in male) (Fig. 9C) with posterior edge triangularly emarginate medially and largely and triangularly emarginate on both sides, lateral emargination about twice as deep as middle one, the protuberances between middle and lateral emarginations acute, exceeding the rounded apices of apicolateral angles. Internal genitalia (Fig. 6C): diverticulum hardly narrowed apically and rounded at apex, about twice as long as its maximal width; spermatheca expanded apically.

#### Distribution.

China (Shaanxi, Gansu, Henan, Anhui, Zhejiang, Hubei, Jiangxi, Hunan, Fujian, Hainan, Guangxi, Sichuan, Guizhou).

### Themus (Telephorops) crassimargo

Taxon classificationAnimaliaColeopteraCantharidae

Champion, 1926

60F01412-C606-574A-A4C4-8CA3F3E05F6C

Figs 3B , 4A, B , 9D

Themus crassimargo Champion, 1926: 127.
Themus (Tryblius) crassimargo : Wittmer 1975: 251, fig. 2 (aedeagus illustration).
Themus (Telephorops) subcaeruleiformis Wittmer, 1983b: 199, fig. 3, 3a (aedeagus illustration), syn. nov.
Themus (Telephorops) crassimargo : Okushima 1999: 59, figs 11 (habitus photo), 35 (female abdominal sternite VIII illustration).

#### Type material examined.

1♂ (BMNH, **lectotype** of *Themus crassimargo*), [p]“Gopaldhara, Sikkim, vii.1924, H. Stevens”, [p] “LECTOTYPUS”, [p] “Themus \ crassimargo \ Champ.”. The lectotype was designated by Wittmer (1975).

1♂ (NHMB, holotype of Themus (Telephorops) subcaeruleiformis), [h] “Kuanshien (Guanxian, now is Dujiangyan) \ Umg. VIII.1934”, “600–1300m \ Szechwan (Sichuan), China”, [p] “HOLOTYPUS”, [h] “T. (Telephorops) subcaerulei- \ formis Wittm. \ det. W. Wittmer”, [p] “Naturhist. \ Museum Basel \ coll. W. Wittmer”, [p] “CANTHARIDAE \ CANTH00001233”.

#### Other material examined.

**CHINA**, **Xizang**: 1♂ (CAUB), Zayü, Shajiong, 1700 m, 1978.VI.26, leg. F.S. Li. **Sichuan**: 1♀ (IZAS), 70 km West Chengdu, Qingcheng, Hou Shan mts., 1360 m, 30°44'N, 103°08'E, 2004.VIII.28, S. Murzin.

#### Distribution.

China (Xizang, Sichuan), N. India, Bhutan, Nepal.

#### Supplementary description.

Female (Fig. 3B). Like male, but antennomeres V–XI without impressions along outer edges (while present with smooth narrow longitudinal or oblong impressions in male), terminal abdominal ventrite wide (while narrow and triangular in male) (Fig. 9D) with posterior edge triangularly protuberant on each side, space between lateral protuberances about twice as wide as each width.

#### Remarks.

Themus (T.) subcaeruleiformis Wittmer, 1983 was originally described based on a single male type, from China, Szechwan, Kuanshien Umg. (now in Sichuan, Dujiangyan). Here a female (Fig. 3B) collected from Qingcheng, which is near the type locality, is discovered for the first time. The structure of its abdominal sternite VIII (Fig. 9D) is like that of *T. crassimargo* Champion, 1926, illustrated by Okushima (1999: fig. 35). Furthermore, *T. subcaeruleiformis* was only compared with *T. subcaeruleus* located in Yunnan, China in the original publication (Wittmer 1983b), but not with species from the Himalayas (Wittmer 1975).Moreover, the types of *T. subcaeruleiformis* and *T. crassimargo* were compared, but no differences between them were found in external morphology (Fig. 4A, B) and aedeagi illustrated by Wittmer (1975: fig. 2; 1983b: fig. 3, 3a).Therefore, *T. subcaeruleiformis* is proposed here to be junior synonym of *T. crassimargo*, according to the Principle of Priority (ICZN 1999, Article 23.1).

### Themus (Telephorops) crassipes

Taxon classificationAnimaliaColeopteraCantharidae

Pic, 1929

1E44617C-528A-5E53-9713-27E740C273BF

Figs 5A–C , 6D , 9E

Themus crassipes Pic, 1929b: 8.
Themus (Telephorops) crassipes : Wittmer 1983b: 191, 200, fig. 64 (female abdominal sternite VIII illustration).

#### Type material examined.

1♂ (MNHN, holotype), [p]“CHAPG. prov. De \Laokay. Ht.-Tonkin”, [h]“*Themus*\ *crassipes* \ n. sp.”, [h]“type”, [p]“HOLOTYPUS”, [h]“*Themus* \ (*Telephorops*)\*crassipes* \ Pic\ det. W. Wittmer”.

#### Other material examined.

**CHINA, Guangxi**: 1♂, 1♀ (IZAS), Leye, Yachang Forestry, Nanchao, 1130 m, 2004.VII.26, leg. X. Yu; 1♀ (MHBU), Tianlin, Cenwanglaoshan, 2014.VIII.16, leg. J.H. Huang; 1♂ (IZAS), Jinxiu, Rd. Jinzhong, 1100 m, 1999.V.12, leg. X.K. Yang; 2♀ (IZAS), same locality, 1000 m, leg. X. Z. Zhang.

#### Supplementary description.

Male. Aedeagus (Fig. 5A–C): ventral process of each paramere about 4 times as long as wide in ventral view, expanded and nearly globose at apex in lateral view; conjoint dorsal plate of parameres not reaching apices of ventral processes, depth of middle emargination about two-fifths of entire length.

Female. Like male, but antennomeres V–X without impressions along outer edges (while present with smooth narrow longitudinal or oblong impressions in male), terminal abdominal ventrite wide (while narrow and triangular in male) (Fig. 9E) with posterior edge narrowly and triangularly emarginate medially between paired rounded protuberances, each protuberance nearly as wide as space between it and apicolateral angle and hardly exceeding apex of apicolateral angle. Internal genitalia (Fig. 6D): diverticulum hardly narrowed apically and rounded at apex, about 2.5 times as long as its maximal width; spermatheca expanded apically.

#### Distribution.

China (Guangxi, Yunnan); Vietnam.

### Themus (Telephorops) impressipennis

Taxon classificationAnimaliaColeopteraCantharidae

(Fairmaire, 1886)

8A811604-CDDC-5E2F-B21C-4144A0D766A1

Figs 5D–F , 7A , 9F

Telephorops impressipennis Fairmaire, 1886: 339.
Telephorops violaceipennis Gorham, 1889: 105. Synonymized by Wittmer 1983b: 199.
Themus (Telephorops) impressipennis : Wittmer 1983b: 199, fig. 60 (female abdominal sternite VIII illustration); 1983a: 153, fig. 51a (female abdominal sternite VIII illustration).

#### Type material examined.

1♂ (MNHN, **holotype** of *Telephorops impressipennis*), [h]“KouyTcheou (China, **Guizhou**)”, [h]“*Telephorops* \ *impressipennis* \ Fairm.”, [p]“HOLOTYPUS”, [h] “*Themus* \ (*Telephorops*) \ *impressipennis* \ (Fairm.) \ det. W. Wittmer”.

1♀ (MNHN, **holotype** of *Telephorops violaceipennis*), [p]“Kiukiang (China, **Jiangxi**) \ June 1887\ A.E. Pratt”, [h]“Type”, [h]“*violaceipennis*”, [h]“*Themus* \ (*Telephorops*) \ *impressipennis* \ (Fairm.) \ det. W. Wittmer”.

#### Other material examined.

**CHINA**, **Guizhou**: 2♀ (MHBU), Daozhen, Xiannvhe, 2004.VIII.24–26, leg. X.J. Yang & H.R. Hua; 1♀ (MHBU), Suiyang, Baishaogou, 2010.VIII.14, leg. L.Y. Guo; 1♂ (IZAS), Fanjingshan, Huguosi, 1350 m, 2001.VIII.3, leg. Q.Z. Song; 1♀ (IZAS), Fanjingshan, Heihaihe, 500 m, 2001.VII.27, leg. Q.Z. Song; 1♂, 2♀ (NHMB), Dakua, 35 km NE Leishan, 1994.VI.20.–24, lgt. Bolm; 1♂, 1♀ (NHMB), Leigongshan, Xijiang, 1200–1900 m,1997.V.29–VI.2, lgt. Bolm. **Sichuan**: 1♂ (IZAS), Nanyang, 1200 m, 1987.VII. 17, leg. L.L. Yang; 1♀ (IZAS), Emei Shan, Xixiangchi, 550–750 m, 1957.VI.8, leg. F.X. Zhu; 1♀ (NHMB), Kwanhsien, 1928.VII.18,collector unknown; 1♂, 1♀ (NHMB), Chuanxian, 600 m, 1996.VII.12.–14, L. M. Bocák; 1♂, 2♀ (NHMB), Mt. Omei, 6000 ft, 1925.VIII.6.–15, coll. D. C. Graham; 1♀ (NHMB), Guanxian, Dujiangyan Park, 1996.VIII.2, A. Zamotajiov & A. Mirostrikov; 1♀ (NHMB), Guanxian, 1992.VII.8, lgt. R. Dunda; 1♀ (NHMB), Gonggashan, Moxi, 1300 m, 29°13'N, 102°10'E, 1996.VII.10.–11, J. Farkač, P. Kabátek&Smetana; 1♂ (NHMB), Emei Shan, 2500–1800 m, 1992.VII. **Yunnan**: 1♀ (NHMB),Vallis flumin, Soling-ho., coll. R. Hicker. **Hubei**: 1♂, 3♀ (MHBU), Dabieshan, Taohuachong, 2014.VI.23–27, leg. X.R. Li; 1♂, 2♀ (MHBU), Dabieshan, Wujiashan, 2014.VI.28–30, leg. X.R. Li; 2♀ (MHBU), Wufeng, Houhe, 2002.VII.15, leg. S.X. Zhou; 1♀ (MHBU), same data, leg. Z.L. Xiang; 1♀ (MHBU), same locality, 2002.VII.16, leg. H.M. Zhang; 1♀ (MHBU), same locality, 2002.VII.16, leg. F.Y. Wang; 1♀ (MHBU), same locality, 2002.VII.16, leg. J. Guo; 1♀ (MHBU), same locality, 2002.VII.17, leg. L. Wang; 2♀ (MHBU), same locality, 2002.VII.18, leg. Y. Liu; 1♀ (MHBU), same data, leg. F. P. Fu; 1♀ (MHBU), same locality and collector, 2002.VII.19; 1♀ (MHBU), same data, leg. M. Wang; 2♀ (MHBU), same data, J.B. Yan; 1♀ (MHBU), same locality, 2002.VII.10, S.H. Yu; 1♀ (MHBU), same locality, 2002.VII.20, leg. P.B. Luo; 1♀ (MHBU), same data, leg. H.M. Zhang; 1♀ (MHBU), same data, leg. J.R. Zheng; 1♂ (MHBU), same data, leg. C.H. Shi; 1♂ (MHBU), same data, leg. H.F. Li; 1♀ (MHBU), same locality, 2002.VII.26, leg. P. Shen; 1♂, 1♀ (MHBU), same locality, 2002.VII.14, leg. S.X. Zhou; 1♂ (MHBU), same data, leg. H.F. Li; 1♂ (MHBU), same data, leg. C.H. Shi; 1♂ (MHBU), same locality, 2002.VII.15, leg. X.Q. Yu; 1♂ (MHBU), same locality, 2002.VII.19, leg. F.P. Fu; 1♀ (MHBU), Wufeng, Changleping, 2008.VII.17, leg. H.P. Zhang; 1♀ (MHBU), Xingshan, Gaolan, 1000 m, 2004.VIII.11, leg. P. Jia; 1♀ (MHBU), same data, leg. J. Xu; 1♀ (MHBU), Xingshan, Huangliang, 1000 m, 2004.VII.12, leg. X.G. Zhou; 1♀ (MHBU), same locality, 2004.VII.13, leg. H. Pan; 1♀ (MHBU), same locality, 2004.VII.16, leg. D.W. Chen; 1♂ (MHBU), same locality, 2004.VII.13, leg. Y.P. Zou; 1♀ (MHBU), Xingshan, Nanyang, 1000 m, 2004.VII.13, P.P. Wang; 1♀ (MHBU), same locality, 2004.VII.14, leg. D.X. Tan; 2♀ (MHBU), Shennongjia, Jiuhuping, 1900 m, 2006.VII.29, leg. L.K. Tan; 1♀ (MHBU), Shennongjia, Muyu, 900 m, 2004.VIII.12, leg. Z.X. Liu; 1♀ (MHBU), same data, leg. R.L. Han; 1♂ (MHBU), same locality, 1200 m, 2004.VIII.12, leg. D.Y. Pan; 1♀ (MHBU), Changyang, Tianzhushan, 2005.VII.13, leg. X. Ming; 1♀ (MHBU), same locality, 2005.VII.12, leg. Q. W. Wang; 1♀ (MHBU), Changyang, Langping, Changfeng, 900 m, 2012.VII.4, leg. H. Zheng; 1♀ (MHBU), Badong, Lvcongpo, 1700 m, 2006.VII.14, leg. H.Y. Bao; 1♀ (MHBU), Badong, Tiansanping, 1500 m, 2006.VII.14, leg. Y.L. Chen; 1♂ (MHBU), same data, leg. F. Xia; 1♀ (MHBU), same data, leg. F. Yang; 1♀ (MHBU), Yichang, Xiabaoping, 1000 m, 2004.VIII.11, leg. Q. Xie; 1♀ (MHBU), same data, 2004.VIII.11, leg. W. M. Li; 1♀ (MHBU), same locality, 2004.VIII.13, leg. S.J. Huang; 1♂ (MHBU), same data, leg. J. Li; 1♂ (MHBU), same data, leg. H.Y. Lei; 1♀ (MHBU), same locality, 2004.VIII.14, leg. B.J. Yu; 1♀ (MHBU), Yichang, Dalaoling Forestry, 2010.VI.24, leg. W. Li; 1♀ (MHBU), Yichang, Xianrenxi, 2009.IX.12, leg. G.L. Xie; 1♂ (MHBU), same locality and collector, 2009.VI.25; 1♂ (IZAS), Xingshan, Longmenhe, 1350 m, 1993.VII.18, leg. B.W. Sun; 1♀ (IZAS), same data, 1993.VII.14; 1♂ (NHMB), Lichuan, Shaoho, 1948.VIII.13, coll. Gressitt & Djou; 1♂ (NHMB), same data, 1948.VIII.12; 1♂ (NHMB), same data, 1948.VIII.24; 1♂ (NHMB), same data, 1948.VIII.26. **Shaanxi**: 1♀(MHBU), Meixian, Songping, 2012.VII.12,leg. G.D. Ren; 1♂(MHBU), Nanzheng, Beiba, 2005.VI.19–22, leg. Y.B. Ba; 1♀ (MHBU), Chushui, Niubeiliang, 2011.VIII.22–29, leg. X.C. Zhu & Y.C. Zhao; 1♂, 1♀ (IZAS), Ningshan, Huoditang, 1580–1650 m, 1999.VI.27, leg. D. C. Yuan. **Gansu**: 1♂ (IZAS), Kangxian, Qinghelinchang, 1400 m, 1998.VII.8, leg. J. Yao; 1♀ (IZAS), Kangxian, Douba, 1050m, 1999.VII.6, leg. H. J. Wang. **Henan**: 1♂ (IZAS), Songxian, Baiyunshan, 1600 m, 2002.VII.19, leg. W.Z. Li; 1♀ (IZAS), Lushixian, Jihelinchang, 1200 m, 2001.VII.20, leg. K.Z. Dong. **Hunan**: 1♀ (MHBU), Changsha Agriculture University, 2012.VII.23, leg. H. Xu; 1♂ (IZAS), Yongshun, Shanmuhe Forestry, 600–820 m, 1988.VIII.7, leg. S.Y. Wang; 1♀ (IZAS), Sangzhi, Tianpingshan, 1370–1570 m, 1988.VIII.13, leg. S.Y. Wang; 1♀ (NHMB),Wulingshan, Tianzishan Nat. Res., 800 m, 1997.VI.16.–18, lgt. Bolm; 1♀ (NHMB), Kiang Jia Jie, 1200–1600 m, 1992.VII. **Zhejiang**: 1♀(MHBU), Hangzhou, Lin’an, Dajingwu, 2012.V.10, leg. H. Xu; 4♂, 1♀(MHBU), Lin’an, Qingliangfeng, Shunxi, 2012.VI.25, leg. H. Xu; 1♂, 1♀(MHBU), Longquan, Fengyangshan, 2007.VII.29, leg. L.K. Tan; 1♀(MHBU), same locality, 2007.VII.27, leg. G.L. Xie; 1♀ (MHBU), same locality and collector, 2007.VII.26; 1♂ (IZAS), Tienmushan, 1935.VII.15, collector unknown; 1♀ (IZAS), same data, 1935.VIII.4. **Jiangxi**: 2♂ (NHMB), Kuling, 1934.IX.4, coll. O. Piel. **Taiwan**: 1♀(NHMB), Formosa, T. Kano. **Fujian**: 1♀ (MHBU), Wuyishan, Tongmu, Tongmuguan-Sangang, 740–1160 m, 2004.VIII.20, leg. D.K. Zhou; 1♀ (IZAS),Dehua, Lishan, 900–1200 m, 1960.VI.12, leg. F.J. Pu; 1♀ (IZAS), Jiangle, Longqishan, 1991.V.25, leg. Y. S. Shi; 2♂ (NHMB), Kuatun, 1946.IX.18. **Anhui**: 6♂, 4♀ (MHBU), Shexian, Qingliangfeng, 2013.VI.5–9, leg. J.S. Xu & C.X. Yuan; 2♂ (NHMB), Kiuhua Shan, 1932.IX, G. Liu Fukien; 1♂, 2♀ (NHMB), Yuexi, Miaodaoshan mts., 600–1300 m, 30°48'N, 116°05'E, 1995.VII.18.–20, lgt. L. R. Businský. **Guangdong**: 1♀ (MHBU), Nanling, 2010.VIII.10, leg. H.Y. Liu. **Guangxi**: 1♀ (MHBU), Jiuwandashan, Jiuren Reserve Station, 2003.VIII.3, leg. L.L. Zhang; 1♂, 1♀ (IZAS), Longsheng, Tianpingshan, 740 m, 1963.VI.17, leg. S.Y. Wang.

#### Supplementary description.

Male. Aedeagus (Figs 5D–F): ventral process of each paramere about 3 times as long as wide in ventral view, expanded and nearly globose at apex in lateral view; conjoint dorsal plate of parameres hardly shorter than ventral processes, depth of middle emargination about one-third of entire length.

**Figure 7.**
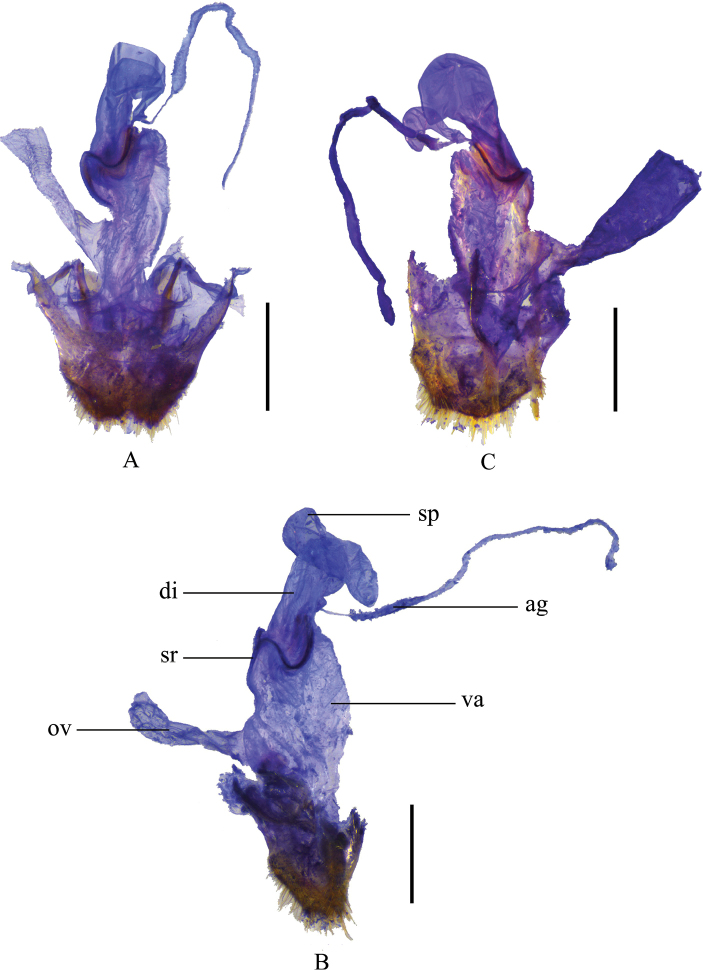
Female internal genitalia, lateral view **A***Themus impressipennis* (Fairmaire, 1886) **B***T. laboissierei* (Pic, 1921) **C***T. masatakai* Okushima, 2003. Scale bars: 1.0 mm (sp: spermatheca; sr: sclerotized ring; va: vagina; di: diverticulum; ov: median oviduct; ag: accessory gland).

Female. Like male, but antennomeres V–X without impressions along outer edges(while present with smooth narrow longitudinal or oblong impressions in male), terminal abdominal ventrite wide (while narrow and triangular in male) (Fig. 9F) with posterior edge narrowly and triangularly emarginate medially and paired rounded protuberances, each nearly as wide as the distance between it and apicolateral angle and not reaching apex of the latter. Internal genitalia (Fig. 7A): diverticulum little thinned apically and rounded at apex, about 2.5 times as long as its maximal width; spermatheca expanded apically.

#### Distribution.

China (Gansu, Shaanxi, Henan, Jiangsu, Anhui, Zhejiang, Hubei, Jiangxi, Hunan, Fujian, Taiwan, Guangxi, Sichuan, Guizhou, Yunnan).

### Themus (Telephorops) laboissierei

Taxon classificationAnimaliaColeopteraCantharidae

(Pic, 1929)

C67A9CCF-88D3-54BE-B696-EB0A614D959F

Figs 4C–D , 7B , 9G

Triblius laboissierei Pic, 1929a: 195, 196.
Themus (Tryblius) separandus Wittmer, 1975: 252, fig. 3 (aedeagus illustration). **syn. n.**
Themus (Telephorops) laboissierei : Wittmer 1983b: 200, figs 4 (aedeagus illustration), 65 (female abdominal sternite VIII illustration).
Themus (Telephorops) separandus : Kazantsev and Brancucci 2007: 271 (distributional data).

#### Type material examined.

1♂ (MNHN, **lectotype** of *Triblius laboissierei*), [p-h]“TONKIN \ Chapa \ 3.VII.1917 \ JEANVOINE”, [h]“*Tryblius* \ *laboissierei* \ n. sp.”, [h] “type”, [p]“LECTOTYPUS”, [h]“Themus \ (Telephorops) \ laboissierei \ Pic \ det. W. Wittmer”. The lectotype was designated by Wittmer (1983b).

1♂ (BMNH, **holotype** of Themus (Telephorops) separandus), [p] “Gopaldhara, Darjeeling, 25.VII.1914, 3440–4720', leg. H. Stevens”, [p] “HOLOTYPUS”, [h] “Themus \ (Tryblius) \ separandus \ Wittm. \ det. W. Wittmer”.

#### Other material examined.

**CHINA**, **Yunnan**: 1♂ (IZAS), Jinping, Hetouzhai, 1700m, 1956.V.9, leg. K.R. Huang; same data, 1500–1700 m, 1956.V.11; 1♂, 1♀ (IZAS), Xishuangbanna, Menghai, 1200–1600 m, 1958.VII.18, leg. S.Y. Wang; 1♂ (IZAS), same locality, 1958.VII.21, leg. F.J. Pu;1♂ (MHBU), Qushi, Jiangmu, 2011.VII.16,leg. H.Y. Liu. **Guangxi**: 1♂, 3♀ (MHBU), Tianlin, Cengwanglaoshan, 2014.VIII.16, leg. J.H. Huang; 1♀ (MHBU), same locality, 1300–1400 m, 2009.V.16–19, collector unknown.

#### Supplementary description.

Female. Like male, but antennomeres V–X without impressions along outer edges (while present with smooth narrow longitudinal or oblong impressions in male), terminal abdominal ventrite wide (narrower and triangular in male) (Fig. 9G) with posterior edge narrowly and triangularly emarginate medially between paired obtuse protuberances, each protuberance nearly as wide as the distance between it and apicolateral angle and not reaching apex of the latter. Internal genitalia (Fig. 7B): diverticulum hardly narrowed apically and rounded at apex, about three times as long as its maximal width; spermatheca expanded apically.

#### Distribution.

China (Yunnan, Guangxi); northern Laos, northern Vietnam, northern India.

#### Remarks.

Themus (Tryblius) separandus was described based on a single male type and its aedeagus was illustrated by Wittmer (1975). Except the original publication, no additional information was available. The type locality is “Gopaldhara, Darjeeling” (N. India), not Bhutan as that listed by Kazantsev and Brancucci (2007).

Wittmer (1975) noted that the single specimen designated as holotype of *T. separandus* was separated from the collection of *T. crassimargo* in BMNH. Wittmer differentiated *T. separandus* from *T. crassimargo* by the structure of aedeagus, also from *T. cavipennis* and *T. nepalensis* in the body coloration and aedeagus. He made no comparison with other species.

In the present study, the habitus (Fig. 4C–D) and aedeagi of *T. separandus* and *T. laboissierei* were compared (Wittmer 1975: fig. 2; Wittmer 1983b: 4), but no differences found. Thus we recommend *T. separandus* Wittmer, 1975 to be junior synonym of *T. laboissierei*, according to the Principle of Priority (ICZN 1999, Article 23.1).

### Themus (Telephorops) masatakai

Taxon classificationAnimaliaColeopteraCantharidae

Okushima, 2003

9B7F7467-20BC-5BF2-A6AA-5DD9361EE2F1

Figs 7C , 9H

Themus (Telephorops) masatakai Okushima, 2003: 280, figs 1–4 (habitus photo, aedeagus illustrations); Kopetz 2010: 185 (distributional data), fig. 4 (aedeagus illustration); Kopetz 2016: 255, figs 15 (habitus photo), 44 (female abdominal sternite VIII photo).

#### Material examined.

**LAOS**: 1♂, 1♀ (NHMB), Oudomxai, 17 km NEE, 1100 m, 20°45'N, 102°09'E, 2002.V.1.–9, leg. Vit Kubáň; 1♂ (NHMB), Phongsaly, Ban Sano Mai, 1150m, 21°21'N, 102°03'E, 2004.V.19.–26, M. Brancucci; 1♂ (NHMB), Phongsaly, 1500 m, 21°41'N, 102°06'E, 2004.V.6.–17, M. Brancucci; 1♂ (NHMB), 20 km NW Louang Namtha, 900–1000 m, 21°09.2'N, 101°18.7'E, 1997.V.5.–30, C. Holzschuh.

**CHINA**,**Yunnan**: 1♂ (IZAS), Xishuangbanna, Meng’a, 1050–1080 m, 1958.V.13, leg. F.J. Pu; 1♂ (IZAS), same data, leg. S.Y. Wang; 1♀ (IZAS), same locality and collector, 1958.VIII.10; 1♂ (IZAS), Simao, Rd. Kunluo 591 km, 1350 m, 1957.V.11, leg. F.J. Pu; 1♀ (IZAS), Simao, 1957.V.23, leg. A. Мэнцяский; 1♀ (IZAS), Simao, 1200 m, 1957.V.11, leg. S.Y. Wang.

#### Supplementary description.

Female. Like male, but antennomeres VII–XI without impressions along outer edges (while present with smooth narrow longitudinal or oblong impressions in male), terminal abdominal ventrite wide (while narrow and triangular in male) (Fig. 9H) with posterior edge narrowly and triangularly emarginate medially between paired rounded protuberances, each protuberance nearly as wide as the distance between it and apicolateral angle and exceeding apex of the latter. Internal genitalia (Fig. 7C): diverticulum expanded apically and rounded at apex, about twice as long as its maximal width; spermatheca expanded apically.

#### Distribution.

China (Yunnan, Guangxi); Laos, northernVietnam.

### Themus (Telephorops) minor

Taxon classificationAnimaliaColeopteraCantharidae

Wittmer, 1997

71F6BDD2-7E91-54AD-93E8-4E59B7151C5B

Themus (Telephorops) minor Wittmer, 1997: 272, fig. 104 (aedeagus illustration); Kopetz 2010: 185, fig. 44 (female abdominal sternite VIII illustration).

#### Type specimens examined.

1♂ (**holotype**, NHMB), [p] “YUNNAN, 23.-24.JUN \ YULONG Mts., 1993 \ 27.00N 100.12E \ Bolm lgt. 3200m”, [p] “HOLOTYPUS”, [h] “Th. (Tryblius) \ minor Wittm. \ det. W. Wittmer”, [p] “CANTHARIDAE \ CANTH00001283”.

#### Distribution.

China (Yunnan).

### Themus (Telephorops) nepalensis

Taxon classificationAnimaliaColeopteraCantharidae

(Hope, 1831)

F1AEBBDF-07F1-5924-96D1-4AE0042CC6D3

Telephorus nepalensis Hope, 1831: 26.
Themus (Tryblius) nepalensis : Wittmer 1975: 252.
Themus (Telephorops) nepalensis : Okushima 1999: 58 (distributional data), figs 9 (habitus photo), 31–33 (aedeagus and female abdominal sternite VIII illustrations).

#### Distribution.

Northern India, Nepal.

### Themus (Telephorops) sauteri

Taxon classificationAnimaliaColeopteraCantharidae

(Pic, 1912)

5F90C65F-4048-5CA9-8910-924F8FA09F43

Figs 8A , 9I

Cantharis sauteri Pic, 1912: 46.
Themus sauteri : Wittmer 1954: 276.
Themus (Telephorops) sauteri : Wittmer 1983a: 197, figs 47 (aedeagus illustration), 50 (female abdominal sternite VIII illustration).

#### Material examined.

**Taiwan**: 1♂ (NHMB), Nanshanchi, 1978.VI.18, H. Akiyama; 1♂, 1♀ (NHMB), Formosa, T. Kano; 1♀ (NHMB), Wushe, 1975.VI.9, K. Akiyama; 1♂ (NHMB), Taichung Hsien, Kukuan, 1996.VII.12, leg. C. Lou; 1♂ (NHMB), same data, 1994.VI.20; 1♀ (NHMB), Nantou Hsien, Sungkang, 1995.VII.17, leg. C. Lou; 1♀ (NHMB), Nantou Hsien, Shintzetou, 1994.VII.14, leg. C. Lou; 1♂, 2♀(IZAS), Taichung Hsien, Kukuan,1996.VII.12, leg. C. Lou; 1♀ (IZAS), Mt. Nantou Hsien, Hohwangshan, 1997.VIII. 27, leg. C. Lou.

#### Supplementary description.

**Figure 8.**
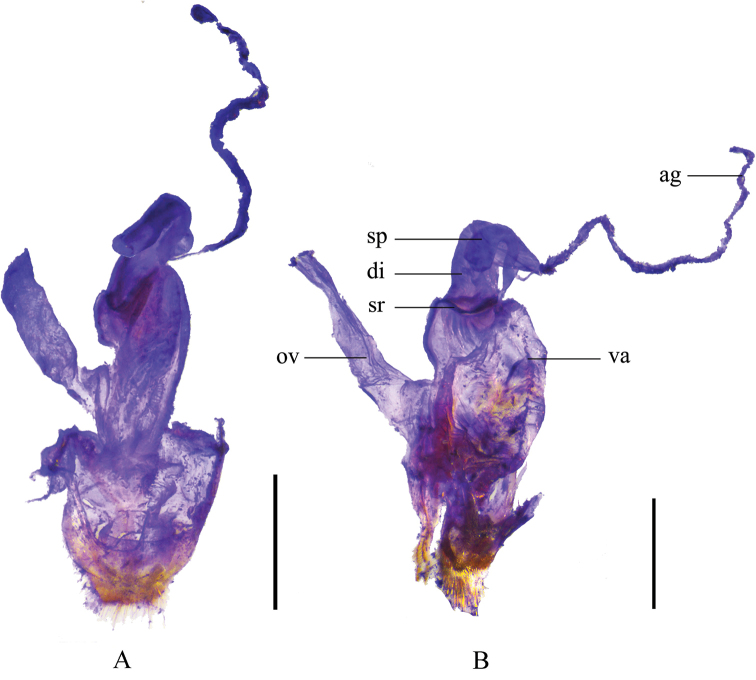
Female internal genitalia, lateral view **A***Themus sauteri* (Pic, 1912) **B***T. uncinatus* Wittmer, 1983. Scale bars: 1.0 mm.

**Figure 9.**
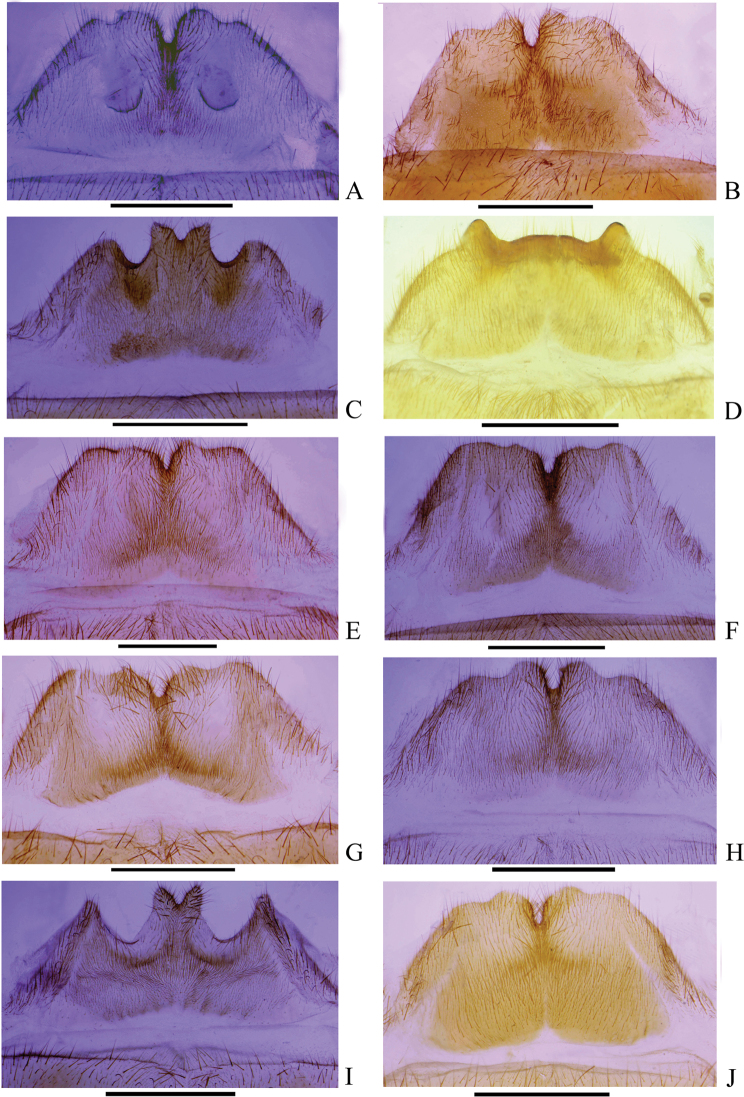
Female abdominal sternite VIII, ventral view **A***Themus bicoloricornis* Wittmer, 1983 **B***T. cavipennis* Champion, 1926 **C***T. coelestis* (Gorham, 1889) **D***T. crassimargo* Champion, 1926 **E***T. crassipes* Pic, 1929 **F***T. impressipennis* (Fairmaire, 1886) **G***T. laboissierei* (Pic, 1921) **H***T. masatakai* Okushima, 2003 **I***T. sauteri* (Pic, 1912) **J***T. uncinatus* Wittmer, 1983. Scale bars: 1.0 mm.

Female. Like male, but antennomeres VI–X without impressions along outer edges (while present with smooth narrow longitudinal or oblong impressions in male), terminal abdominal ventrite wide (while narrow and triangular in male) (Fig. 9I) with posterior edge triangularly emarginate medially and largely and triangularly emarginate on both sides, lateral emargination about 3 times as deep as middle one, the protuberances between middle and lateral emarginations acute, exceeding the acute apices of apicolateral angles. Internal genitalia (Fig. 8A): diverticulum hardly narrowed apically and rounded at apex, about twice as long as its maximal width; spermatheca expanded apically.

#### Distribution.

Taiwan.

### Themus (Telephorops) subcaeruleus

Taxon classificationAnimaliaColeopteraCantharidae

(Pic, 1911)

1CC50AB1-99E5-5129-B282-CBD95BEBBEC0

Tryblius cavipennis var. subcaeruleus Pic, 1911: 132.
Themus (Tryblius) subcaeruleus : Pic 1929a: 195.
Themus (Telephorops) subcaeruleus : Wittmer 1983b: 199, figs 2 (aedeagus illustration), 62 (female abdominal sternite VIII illustration).

#### Type material examined.

1♂ (MNHN, **lectotype**), [p]“Yünan \ China”, [h]“type”, [h]“*Tryblius* \ *subcaeruleus* \ Pic”, [p]“LECTOTYPUS”, [h]“*Themus* \ (*Telephorops*) \ *subcaeruleus* \ Pic\ det. W. Wittmer”. The lectotype was designated by Wittmer (1983b).

#### Other material examined.

**CHINA**: 1♀ (NHMB), Yunnan; 1♂ (NHMB), Pe Yen Tsing;1♀ (NHMB), Tche-Ping-Tcheou.

#### Distribution.

China (Yunnan), northern Vietnam.

### Themus (Telephorops) uncinatus

Taxon classificationAnimaliaColeopteraCantharidae

Wittmer, 1983

67C27E57-09A4-5B60-BBAD-6C61FAB310D8

Figs 8B , 9J

Themus (Telephorops) uncinatus Wittmer, 1983b: 200, figs 5 (aedeagus illustration), 66 (female abdominal sternite VIII illustration).

#### Type material examined.

1♂ (holotype, MNHN), [p] “MUSEUM PARIS \ SE-TSCHOUEN (China, SE. Sichuan)\ ENV DE TA_TSIEN-LOU (Dajianlu, now is Kangding) \ MO-SY-MIEN \ Père AUBERT 1902”, [p] “HOLOTYPUS”, [h] “Themus (Telephorops) \ uncinatus \ Wittm. \ det. W. Wittmer”.

#### Other material examined.

**CHINA**, **Sichuan**: 1♂ (NHMB), Jinfo Shan, 1700–1950m, 29°01'N, 107°14'E, 1998.VI.24.–29, D. Král; 1♀ (NHMB), Chadiping, 1200–1500m, 1996.VIII.5.–7, A. Miroshnikov & A. Zamatajiov; 1♂, 1♀ (IZAS), Luding, Moxi, 1500 m, 1983.VI.17, leg. S.Y. Wang; 1♂ (IZAS), Emei Shan, Jiulaodong, 1800–1900 m, 1957.VII.28, leg. K.R. Huang. **Yunnan**: 4♂, 1♀ (MHBU), Lushui, Laowo, 1500 m, 2008.VII.26.–28, leg. J.S. Xu & Z.H. Gao; 1♂, 2♀ (MHBU), Lushui, Pianma, 2005.VII.22.–23, leg. B.Y. Mao & J.S. Xu; 1♀(MHBU), Longling, Longxin, Heishan, 2008.XII.22.–23, leg. J.S. Xu & Z.H. Gao.

#### Supplementary description.

Female. Like male, but antennomeres V–X without impressions along outer edges (while present with smooth narrow longitudinal or oblong impressions in male), terminal abdominal ventrite wide (while narrow and triangular in male) (Fig. 9J) with posterior edge narrowly and triangularly emarginate medially between paired protuberances, each protuberance wider than the distance between it and apicolateral angle and hardly exceeding apex of the latter. Internal genitalia (Fig. 8B): diverticulum narrowed apically and pointed at apex, about twice as long as its maximal width; spermatheca moderately expanded apically.

#### Distribution.

China (Sichuan, Yunnan), northern Vietnam.

## Supplementary Material

XML Treatment for Themus (Telephorops) nepalensis

XML Treatment for Themus (Telephorops) bicoloricornis

XML Treatment for Themus (Telephorops) cavipennis

XML Treatment for Themus (Telephorops) coelestis

XML Treatment for Themus (Telephorops) crassimargo

XML Treatment for Themus (Telephorops) crassipes

XML Treatment for Themus (Telephorops) impressipennis

XML Treatment for Themus (Telephorops) laboissierei

XML Treatment for Themus (Telephorops) masatakai

XML Treatment for Themus (Telephorops) minor

XML Treatment for Themus (Telephorops) nepalensis

XML Treatment for Themus (Telephorops) sauteri

XML Treatment for Themus (Telephorops) subcaeruleus

XML Treatment for Themus (Telephorops) uncinatus
